# Frequency of Depression and Anxiety in Patients With Lung Cancer Undergoing Chemotherapy: A Single-Center, Cross-Sectional Study From Vietnam

**DOI:** 10.7759/cureus.68647

**Published:** 2024-09-04

**Authors:** Vo Thi Ngoc Han, Huynh Thuy Vy, Ho Tat Bang, Lam Quoc Trung, Tran Thanh Vy

**Affiliations:** 1 Department of Infection Control, Cho Ray Hospital, Ho Chi Minh City, VNM; 2 Faculty of Public Health, University of Medicine and Pharmacy at Ho Chi Minh City, Ho Chi Minh City, VNM; 3 Department of Thoracic and Vascular Surgery, University Medical Center Ho Chi Minh City, University of Medicine and Pharmacy at Ho Chi Minh City, Ho Chi Minh City, VNM; 4 Department of Health Management, Faculty of Public Health, University of Medicine and Pharmacy at Ho Chi Minh City, Ho Chi Minh City, VNM; 5 Department of Chemotherapy, University Medical Center Ho Chi Minh City, University of Medicine and Pharmacy at Ho Chi Minh City, Ho Chi Minh City, VNM; 6 Department of Thoracic and Cardiovascular Surgery, Faculty of Medicine, University of Medicine and Pharmacy at Ho Chi Minh City, Ho Chi Minh City, VNM

**Keywords:** hospital anxiety and depression scale, lung cancer, chemotherapy, anxiety, depression

## Abstract

Background

Chemotherapy is a crucial component of multimodality treatment for lung cancer patients. Although considered a vital and effective approach, chemotherapy can induce adverse effects, negatively impacting patients’ quality of life. These adverse effects can also lead to psychological issues, particularly depression and anxiety. This study aimed to determine the frequency of depression, anxiety, and their associated factors in patients with lung cancer undergoing chemotherapy.

Methodology

A cross-sectional study was conducted on 92 patients with lung cancer receiving chemotherapy at the University Medical Center Ho Chi Minh City, Vietnam, from February to May 2023. The patients’ depression and anxiety were assessed using the Hospital Anxiety and Depression Scale.

Results

Among 92 patients with a mean age of 60.4 ± 9.6 years and 58 males (63%), the prevalence of depression was 44.5% (41 patients), with significant associations between depression and gender, economic status, smoking status, and performance status. The prevalence of anxiety was 38% (35 patients), with significant associations between anxiety and gender, chemotherapy cycle, cancer stage, and performance status.

Conclusions

Our study highlights the importance of a multidisciplinary approach to cancer care, ensuring that lung cancer patients receive the necessary support to manage both physical and mental health challenges throughout chemotherapy.

## Introduction

Lung cancer poses a significant burden on healthcare systems worldwide, accounting for approximately 1.8 million deaths annually [1]. Chemotherapy is a crucial component [2] of multimodality treatment for lung cancer patients. While it is considered a vital and effective approach, chemotherapy can induce adverse effects such as alopecia, nausea and vomiting, anorexia, fatigue, and immune system suppression, which can negatively impact patients’ quality of life and treatment adherence [3-5]. In addition to physical impacts, chemotherapy can also lead to psychological issues, particularly depression and anxiety.

A long-lasting low mood or loss of pleasure or interest in activities characterizes depression [6]. Anxiety is a natural stress response. Both disorders share similar symptoms, including fatigue, sleep disturbance, appetite changes, and difficulty concentrating [6-8]. When depression occurs in cancer patients, it adds to their hardship and suffering, even driving some to consider death. A systematic review of 62 studies involving 46,952,813 individuals showed that cancer patients had nearly twice the suicide mortality rate of the general population (standardized mortality ratio = 1.85, 95% confidence interval (CI) = 1.55-2.2). Poor prognosis, advanced stage, and the first year after diagnosis were identified as risk factors [9]. Additionally, a study involving 256 hospitalized lung cancer patients at Bach Mai Hospital in Vietnam found that 8.2% had suicidal ideation two to five times per week [9]. Consequently, depression and anxiety in lung cancer patients undergoing chemotherapy remain a significant challenge in Vietnam, given their high prevalence and mortality rates [10].

In Vietnam, while previous research has explored mental health and quality of life in lung cancer patients in general, studies specifically focusing on patients undergoing chemotherapy remain limited. Despite the high volume of chemotherapy treatments administered daily at the University Medical Center (UMC), no research has yet examined the specific aspects of depression and anxiety in this patient population. Therefore, this study aims to assess the prevalence of depression and anxiety among lung cancer patients undergoing chemotherapy and identify associations between them.

## Materials and methods

Study objectives

This study aimed to determine the prevalence of depression, anxiety, and their associated factors in patients with lung cancer undergoing chemotherapy.

Study design

A cross-sectional study was conducted on patients with lung cancer undergoing chemotherapy at the University Medical Center Ho Chi Minh City (UMC HCMC), Vietnam, from February 2023 to May 2023.

Study participants, sample size, and sampling

The inclusion criteria for the study were patients diagnosed with lung cancer and undergoing chemotherapy. Patients over the age of 18 years who could make their own decisions and agreed to participate in the study were included. Patients who were unable to communicate were excluded, for example, those with speech/hearing disabilities, dementia, or taking antidepressants.

The study employed a convenience sampling method and continued sampling until saturation was reached using the daily admission list of the chemotherapy department at the UMC. After explaining the research objectives and confidentiality to patients, ensuring their understanding of the benefits and risks, their consent was obtained. Face-to-face interviews were conducted in conjunction with a medical record review. The average interview duration was 15-20 minutes.

We applied a formula to estimate a sample size for a proportion, with p of 0.397 representing the rate of depression among patients with non-small-cell lung cancer, based on the study by Tai et al. [11]. We set the margin of error (d) at 0.1 and the value for the desired confidence level at 1.96 for 95% confidence (Z_1-α/2_) = 1.96. Consequently, we calculated the required sample size to be 92 patients.

Data collection and tool

The Hospital Anxiety and Depression Scale (HADS) developed by Zigmond and Snaith in 1983 [12] was used in our study. This 14-item, self-rated scale assesses anxiety (HADS-A) and depression (HADS-D) on a four-point scale ranging from 0 to 3. Each subscale comprises seven items, with scores ranging from 0 to 21, which are mapped to a Likert scale for severity classification into categories such as normal, possible symptoms, and disorder. The Vietnamese version, published by Hanh et al. demonstrated good internal consistency, with Cronbach’s alpha values of 0.80 for the depression subscale and 0.85 for the anxiety subscale [13]. We obtained permission from the original authors to use HADS in our study, ensuring compliance with copyright requirements.

The study explored demographics (age, sex, income, ability to pay) and disease factors (smoking, chemotherapy cycles, cancer stages, performance status) in <20-minute interviews and medical record reviews. Participants were categorized into age groups (<45, 45-60, >60 years), sexes (male, female), economic status (affluent, sufficient, difficult), and ability to pay (yes, no; assessment based on the patient’s feelings during the interview showing their capability of self-payment, meaning they do not need external support beyond their family, such as assistance from relatives, friends, or social support organizations). Disease status variables included smoking status (yes, no), chemotherapy cycles (<5, 5-10, >10), cancer stages (0 to IV), and performance status (PS 1: normal activities, PS 2: tired but with little limitation on physical activity, PS 2: lying in bed <50% of the day, PS 3: lying in bed >50% of the day, PS 4: bedridden; the index assessing the level of patient activity and their ability to self-care, perform daily activities, and maintain physical capability).

Statistical analysis

Data analysis involved EpiData entry and Stata 17.0 (StataCorp., College Station, TX, USA). Binary/qualitative variables were presented as frequencies/percentages, while quantitative variables used means/standard deviations (SDs) (or medians and interquartile ranges for abnormal distributions). Chi-square tests assessed associations between binary independent and outcome variables, with Fisher’s exact test as an alternative when expected values were low. Univariable logistic regression identified factors associated with depression and anxiety (p < 0.05, 95% CI excluding 1). The prevalence ratio (PR) was used to evaluate the magnitude of the association.

## Results

During the study, we recruited 92 lung cancer patients undergoing chemotherapy at the UMC who met the inclusion criteria of the study.

Characteristics of study participants

The demographic and clinical characteristics of the study participants are detailed in Table 1. The mean age was 60.4 years (SD = ±9.6), with age ranging from 32 to 79 years. The majority of the participants were male (63%). Regarding economic status, 73.9% of the patients were classified as having an average economic status, while 10.9% were considered low-income. A significant proportion (88%) of the participants could afford treatment costs. Smoking status was divided, with 51.1% being non-smokers and 46.7% smokers. The most prevalent cancer stage among the participants was Stage IV (64.1%). In terms of performance status, 54.4% reported limited activity levels with low fatigue.

**Table 1:** General characteristics of study participants (n = 92).

Characteristics	Frequency (n)	Ratio (%)
Sex		
Male	58	63
Female	34	37
Age group (years)		
<45	7	7.6
45–60	35	38
> 60	50	54.4
Economic status		
Affluent	14	15.2
Sufficient	68	73.9
Low income	10	10.9
Ability to pay		
Yes	81	88
No	11	12
Smoking status		
Yes	45	48.9
No	47	51.1
Chemotherapy cycle		
<5 chemotherapy doses	52	56.5
5–10 chemotherapy doses	29	31.5
>10 chemotherapy doses	11	12
Cancer stage		
Stage I, II	12	13.1
Stage III	21	22.8
Stage IV	59	64.1
Performance status (PS)		
PS 0	24	26.1
PS 1	50	54.4
PS 2	11	11.9
PS 3, 4	7	6.5

The HADS score

Our study found that 22.8% of the patients exhibited symptoms of depression, and 18.5% had symptoms of anxiety. The actual prevalence rates of depression and anxiety were 21.7% and 19.5%, respectively. Of those, the results identified 14 (15.2%) patients diagnosed with both depression and anxiety (Table 2).

**Table 2:** The Hospital Anxiety and Depression Scale (HADS) scores (n = 92).

HADS score	Depression	Anxiety
Normal (0–7 scores)	51 (55.5%)	57 (62%)
Borderline abnormal (8–10 scores)	21 (22.8%)	17 (18.5%)
Abnormal (11–21 scores)	20 (21.7%)	18 (19.5%)

Factors associated with lung cancer during chemotherapy

Table 3 presents the factors associated with depression and anxiety among the patients. Results revealed that age, insurance status, ability to pay, and cancer stage were not significantly associated with either depression or anxiety. However, gender exerted a significant role, with female patients having a lower risk of both depression (odds ratio (OR) = 0.19) and anxiety (OR = 0.34). Socioeconomic factors were also influential; patients with lower income and those experiencing difficulties in affording treatment had higher risks of depression (OR = 2.53) and anxiety (OR = 2.83). Smoking status (OR = 3.13) and undergoing a higher number of chemotherapy cycles (5-10 cycles; OR = 2.56) were identified as significant risk factors for both conditions. Performance status emerged as a critical factor, with patients having a poor performance status (PS = 0) showing significantly higher risks of depression (OR = 8.57) and anxiety (OR = 5.14) compared to those with a better performance status.

**Table 3:** Relationship between depression, anxiety, and some characteristics of personal diseases (n = 92). ^a^: Chi-square test; ^b^: Fisher’s exact test

Characteristics		Depression				Anxiety			
		Yes, n (%)	No, n (%)	P-value	PR (95% CI)	Yes, n (%)	No, n (%)	P-value	PR (95% CI)
Sex	Male	18 (31.1)	32 (94.1)	0.048^a^	0.19 (0.05–0.77)	15 (25.9)	43 (74.1)	0.047^a^	0.34 (0.11–1.09)
	Female	2 (5.9)	40 (68.9)			3 (8.8)	31 (91.2)		
Economic status	Affluent	1 (7.1)	13 (92.9)	-	1	2 (14.3)	12 (85.7)	-	1
	Sufficient	14 (20.6)	54 (79.4)	0.006^a^	2.53 (1.30–4.93)	11 (16.2)	57 (83.8)	0.862^a^	1.13 (0.28–4.59)
	Difficult	5 (50)	5 (50)	0.006^a^	6.40 (1.69–24.30)	5 (50)	5 (50)	0.087^a^	3.50 (0.84–14.67)
Ability to pay	Yes	15 (18.5)	66 (81.5)	0.057^b^	1	13 (16.1)	6 (54.5)	0.036^b^	
	No	5 (45.5)	6 (54.5)		2.45 (1.11–5.4)	5 (45.5)	68 (83.9)		2.83 (1.25–6.41)
Smoking status	Yes	15 (33.3)	30 (66.7)	0.016^a^	3.13 (1.24–7.95)	11 (24.4)	34 (75.6)	0.248^a^	1.64 (0.69–3.86)
	No	5 (10.6)	42 (89.4)		1	7 (14.9)	40 (85.1)		1
Chemotherapy cycle	<5 chemotherapy doses	9 (17.3)	43 (82.7)	-	1	7 (13.5)	45 (86.5)	-	1
	5–10 chemotherapy doses	8 (27.6)	21 (72.4)	0.278^a^	1.59 (0.69–3.70)	10 (34.5)	19 (65.5)	0.031^a^	2.56 (1.09–6.04)
	>10 chemotherapy doses	3 (27.3)	8 (72.7)	0.434^a^	1.58 (0.50–4.92)	1 (9.1)	10 (90.9)	0.701^a^	0.68 (0.09–5.00)
Cancer stage	I, II	2 (22.2)	10 (83.3)	0.776^a^	0.82 (0.21–3.22)	2 (22.2)	10 (83.3)	0.777^a^	1.23 (0.29–5.12)
	III	6 (28.6)	15 (71.4)	0.432^a^	1.4 (0.60 –3.28)	8 (38.1)	13 (61.9)	0.017^a^	2.81 (1.20–6.56)
	IV	12 (20.3)	47 (79.7)	-	1	8 (13.6)	51 (86.4)	-	1
Performance status	PS 0	2 (8.3)	22 (91.7)	-	1	2 (8.3)	22 (91.7)	-	1
	PS 1	10 (20)	40 (80)	0.235^a^	2.4 (0.57 –10.19)	9 (18)	41 (82)	0.301^a^	2.16 (0.50–9.31)
	PS 2	3 (27.3)	8 (72.7)	0.159^a^	3.27 (0.63–17.04)	4 (36.4)	7 (63.6)	0.062^a^	4.36 (0.93–20.53)
	PS 3,4	5 (72.4)	2 (28.6)	0.003^a^	8.57 (2.08– 35.28)	3 (42.9)	4 (57.1)	0.043^a^	5.14 (1.05–25.15)

## Discussion

This cross-sectional study was conducted on 92 lung cancer patients undergoing chemotherapy at the UMC HCMC, a tertiary referral hospital in Vietnam. Our findings indicate a significant prevalence of depression and anxiety among this population, underscoring the critical need for psychological support alongside chemotherapy for lung cancer patients.

Our study population had a mean age of 60.4 ± 9.6 years, ranging from 32 to 79 years. The age group of 60-70 years accounted for the majority (54.4%). This result is similar to the study by Quyen [14] on primary lung cancer patients and is consistent with the age of onset of cancer in Vietnam. The gender distribution showed a higher proportion of male patients (63%) compared to female patients (37%), reflecting the higher prevalence in men is attributed to smoking, which is a major risk factor for lung cancer, accounting for up to 90% of cases. Regarding economic status, most patients had sufficient (73.9%) or good (15.2%) financial conditions, and 88% had health insurance coverage for chemotherapy, allowing them to afford treatment costs.

The depression rate in our study was 44.5% based on HADS. This is higher than the study by Arrieta et al. on patients with non-small-cell lung cancer [15]. The difference is that the study only included patients with non-small-cell lung cancer, while our study included patients with lung cancer in general. The study found a statistically significant association between general and disease characteristics of the patients and depression. Specifically, men had a 0.19 times higher prevalence of depression than women, and the difference was statistically significant with p-values of 0.048. The study by Lavdaniti et al. on 76 patients with advanced non-small-cell lung cancer suggests that gender affects the rate of depression [16]. This could be attributed to cultural and social factors where men, often seen as primary breadwinners, may experience higher levels of depression and anxiety about treatment costs and family responsibilities, contributing to higher rates of depression and anxiety. Patients with a moderate living standard had a 2.53 times higher rate of depression than those who were well-off, with a statistically significant p-value of 0.006. Additionally, we found that those with poor performance status (PS 3, 4) had an 8.56 times higher rate of depression than those with no activity limitations (PS 0). This result is similar to the studies by Arrieta et al. [15], and Salvo et al. [17] with 1,439 patients with different advanced cancers.

Of the 92 study participants, 35 (38%) were diagnosed with anxiety. This is a notable finding compared to the study by Arrieta et al. [15], which reported an anxiety rate of 34.1%. This is similarly reflected in the rates of depression between the two studies which may be due to differences in research settings and cultural factors between Mexico and Vietnam. No statistically significant gender difference was found in anxiety rates. However, those who were unable to afford treatment costs had 2.83 times higher odds of anxiety compared to those who could afford it (p = 0.036). This finding has not been reported in other studies, but it is understandable considering that most of our study subjects were of retirement age and financially dependent on their children, making treatment costs a psychological burden. The number of chemotherapy sessions had a significant impact on patients, with those undergoing 5-10 sessions having a higher anxiety rate than those receiving fewer than 5 sessions (p = 0.031). This is consistent with the study by Uyen et al. on fatigue in lung cancer patients receiving chemotherapy [18]. Prolonged chemotherapy accumulation can lead to fatigue, which, in turn, can cause patients to worry about the treatment’s effectiveness. Anxiety was more severe in those with poor performance status (PS 3,4), who had 5.14 times higher odds of anxiety compared to those with good performance status (PS 0) (p = 0.043). This finding is consistent with that of Salvo et al. [17]. This observation underscores the need for comprehensive care approaches that address not only the physical but also the psychological aspects of cancer treatment, particularly for patients with advanced disease stages and reduced physical functioning.

The study employed an appropriate assessment, HADS, clear objectives, and rigorous sampling but was limited by a small sample and cross-sectional design, hindering causal inference and generalizability. A validated Vietnamese version of the scale was used to assess reliability and validity, with Cronbach’s alpha coefficients of 0.80 for anxiety and 0.75 for depression. These coefficients are based on the study by Loans et al. [19] conducted on patients with gastroesophageal reflux disease. Previously, Hanh et al. [13]. demonstrated that Cronbach’s alpha coefficients for both subscales exceeded 0.70, indicating high internal consistency, specifically HADS-D (α = 0.80) and HADS-A (α = 0.85). These findings not only reinforce the reliability but also highlight the validity of HADS within the context of research in Vietnam. Consequently, it aids in more effectively assessing depression and anxiety among the patient population during treatment.

Our study highlights a high prevalence of anxiety and depression among lung cancer patients undergoing chemotherapy, similar to findings from international studies​ [20,21]. This indicates the importance of integrating psychological support into standard cancer treatment protocols to enhance patient outcomes and quality of life. Healthcare managers should prioritize mental health by developing comprehensive care models that address both physical and psychological needs. Factors such as gender, economic status, and performance status should be considered to provide tailored psychological interventions for different patient subgroups throughout their cancer journey.

This study provides valuable insights into the prevalence and associated factors of depression and anxiety among lung cancer patients undergoing chemotherapy in Vietnam. The use of the standardized HADS ensures the reliability and validity of the results, contributing to an appropriate assessment of psychological distress in this population. However, the study is limited by its small sample size and cross-sectional design, which restricts the ability to infer causality and generalize the findings to broader populations.

## Conclusions

Our study highlights the high prevalence of depression and anxiety among lung cancer patients undergoing chemotherapy, with significant associations with gender, economic status, ability to pay, smoking status, chemotherapy cycles, and performance status. This evidence suggests the need for integrated care models that include psychological support to improve the overall well-being and treatment outcomes of lung cancer patients in the UMC HCMC.
